# The Chaperone BAG6 Captures Dislocated Glycoproteins in the Cytosol

**DOI:** 10.1371/journal.pone.0090204

**Published:** 2014-03-03

**Authors:** Jasper H. L. Claessen, Sumana Sanyal, Hidde L Ploegh

**Affiliations:** Whitehead Institute for Biomedical Research, Department of Biology, Massachusetts Institute of Technology, Cambridge, Massachusetts, United States of America; Ecole Polytechnique Federale de Lausanne, Switzerland

## Abstract

Secretory and membrane (glyco)proteins are subject to quality control in the endoplasmic reticulum (ER) to ensure that only functional proteins reach their destination. Proteins deemed terminally misfolded and hence functionally defective may be dislocated to the cytosol, where the proteasome degrades them. What we know about this process stems mostly from overexpression of tagged misfolded proteins, or from situations where viruses have hijacked the quality control machinery to their advantage. We know of only very few endogenous substrates of ER quality control, most of which are degraded as part of a signaling pathway, such as Insig-1, but such examples do not necessarily represent terminally misfolded proteins. Here we show that endogenous dislocation clients are captured *specifically* in association with the cytosolic chaperone BAG6, or retrieved *en masse* via their glycan handle.

## Introduction

Secretory and membrane proteins enter the endomembrane system for the most part by co-translational insertion into the endoplasmic reticulum (ER), an environment dedicated to their folding at the hands of specialized chaperones. In the ER the polypeptide backbone is often further modified by co- and post-translational modifications such as glycosylation and the formation of disulfide bonds, which aid in protein folding and thus function. Proteins undergo quality control in the ER prior to negotiating the endomembrane pathway. Those proteins that do not fold properly, or fail to find necessary binding partners, are sequestered and can be transferred across the membrane of the ER to the cytosol, where they are degraded by the ubiquitin-proteasome system (UPS) [1], [2]. This controlled return of secretory proteins to the cytosol is referred to as dislocation, and provides an exit pathway co-opted by viruses such as polyoma and SV40, and bacterial toxins such as Cholera toxin and Pseudomonas Exotoxin A [3].

The study of dislocation has relied mostly on overexpression of known and purposely designed dislocation substrates. An approach based on overexpression is inherently biased, as only a single substrate is tested at a time. Such proteins, truncated or expressed in the absence of their usual binding partners, will commonly fail quality control in the ER and be targeted for dislocation. The proposed involvement of a novel dislocation component is then validated through sequential testing of additional overexpressed and artificial substrates.

Though informative, the enforced high levels of expression of such substrates unavoidably changes the local environment of the ER. The unfolded protein response (UPR), a stereotypical set of adjustments to prevent toxic accumulation of unfolded protein in the ER, is one such example [4]. However, remodeling of the ER by adjustment of its folding capacity may frustrate analysis of the very process under investigation.

Here we apply a different approach to measure protein dislocation in mammalian cells, one which does not require overexpression of proteins in the ER lumen. We define endogenous dislocation substrates as N-linked glycoproteins that appear in the cytosol in their glycosylated state when the activity of N-glycanase, the enzyme that clips N-linked glycans from glycoproteins that arrive in the cytosol, is blocked. Through enzymatic interference with the UPS, we enrich this protein fraction. Our strategy for retrieval is two-pronged: we recover dislocated proteins either in association with a cytosolic chaperone, for which we chose BAG6 (BAT3/Scythe), or through adsorption on a lectin- agarose matrix. We could retrieve endogenous dislocation substrates with either method. This recovery is blocked by expression of a dominant negative form of the AAA ATPase p97, firmly rooting this protein at the heart of the dislocation reaction. We also show that dithiothreitol (DTT), commonly thought to produce an onslaught of misfolded substrates in the ER through interference with the formation and maintenance of disulfide bonds, does not induce accumulation of newly synthesized glycoproteins in the cytosol, but rather rapidly halts the synthesis of signal-sequence containing proteins.

## Materials and Methods

### Antibodies, Cell Lines, and Reagents

Antibodies against the hemagglutinin (HA) and FLAG epitope tags were purchased from Sigma. The TCRα, Derlin2, and PDI antibodies have been described [5]. We thank N. Erwin Ivessa for generously providing anti-ribophorin antibody. Antibodies against BAG6 and GAPDH were obtained from Abcam.

HEK293T cells were purchased from American Type Culture Collection, and transiently transfected using Trans-IT (Takara Mirus Bio).

Concanavalin A conjugated to sepharose was obtained from Sigma. PNGase F and endoH were purchased from New England Biolabs, dithiothreitol from Sigma. Z-VAD-FMK was obtained from VWR (Supplier: EMD Millipore).

### Constructs

Plasmids encoding Ri332, TCRα, EBV DUB, UBX-EBV DUB, p97wt and p97QQ have been described [6]. cDNA encoding BAG6 was isolated from HEK293T cells using a reverse transcriptase system from Promega, and cloned into pcDNA3.1(+) (Invitrogen).

### Immunoprecipitations, Pulse-Chase Experiments, and SDS-PAGE

Cells were lysed in NP40 lysis buffer (0.5% NP40, 10 mM Tris-HCl, 150 mM NaCl, 5 mM MgCl_2_, pH 7.4) supplemented with a complete protease inhibitor cocktail (Roche). The immunoprecipiation was performed using 30 µl of immobilized rProtein A (IPA 300, Repligen) with the relevant antibodies, or with 12 µl anti-HA Affinity Matrix (3F10, Roche), for 3 h at 4°C with gentle agitation. The lysates were normalized to relative protein concentration prior to incubation with antibodies.

To achieve steady-state protein labelling, cells were incubated overnight with 100 µCi/ml of [^35^S]methionine/cysteine (Perkin Elmer) in methionine/cysteine-free DMEM supplemented with 10% dialyzed IFS at 37°C.

Pulse-chase experiments were performed as described [6]. In short, prior to pulse labelling, the cells were starved for 45 min in methionine/cysteine-free DMEM at 37°C. Cells were then labelled for 10 min at 37°C with 100 µCi/ml of [^35^S] methionine/cysteine. Incorporated radioactivity was quantified after lysis and TCA precipitation using a scintillation counter, in order to equalize for incorporated ^35^S levels. The target protein was isolated through immunoprecipitation, and immune complexes were eluted by boiling in reducing sample buffer, subjected to SDS-PAGE (10%), and visualized by autoradiography. Densitometric quantification of radioactivity was performed on a PhosphorImager.

### Immunoblotting

For immunoblot analysis, cell lysates were prepared by solubilizing cells in 1% SDS, and equivalent amounts of total cellular protein were used for immunoblotting using HRP-conjugated antibodies.

### Cell Fractionation using perfringolysin O

Cells were permeabilized using perfringolysin O toxin from *Clostridium perfringens*. Both the purification of the toxin and the experimental set-up have been described in detail [7]. In short, cells were incubated with 100 nM PFO in Hanks' balanced salt solution (HBSS) on ice. Unbound toxin was removed by washing the cells with cold HBSS, after which the cells were incubated at 37°C to allow pore formation. The cytosolic fraction (supernatant) was collected after centrifugation at 500×*g* for 5 min.

## Results

### Recovery of dislocated glycoproteins through interaction with BAG6

Is it possible to capture dislocated glycoproteins in the cytosol, prior to their degradation by the proteasome? Under normal conditions, dislocation and degradation are tightly coupled, making it difficult to capture transient intermediates. Some dislocation substrates, such as truncated ribophorin (Ri332), accumulate in the cytosol once proteasomal activity is blocked with proteasome inhibitors [8]. Alternatively, during expression of the catalytic domain of the Epstein Barr Virus large tegument protein BPFL1 (EBV DUB), a deubiquitylase (DUB), dislocation continues unabated, but degradation of dislocated substrates ceases [6]. Where a DUB activity is required for the dislocation reaction, preemptive removal of the ubiquitin signal impedes proteasomal targeting and thus destruction of the substrate. For ER-resident dislocation substrates, this latter effect is enhanced when the EBV DUB is targeted to p97 -the motor that drives dislocation- via a UBX-domain [6].

Several dislocated glycoproteins that accumulate in the cytosol are found in complex with the BAG6 protein. BAG6 associates with a number of well-studied components of the dislocation machinery, and directly aids in the dislocation reaction [5], [9]. This association does not depend on a proteasomal blockade, as BAG6 interacts with the alpha chain of the T Cell Receptor (TCRα) even in the absence of EBV DUB expression [5]. BAG6 associates with unstructured polypeptides [6], [10]–[12], and could prevent aggregation of partly unfolded glycoproteins *en route* to the proteasome.

**Figure 1:**
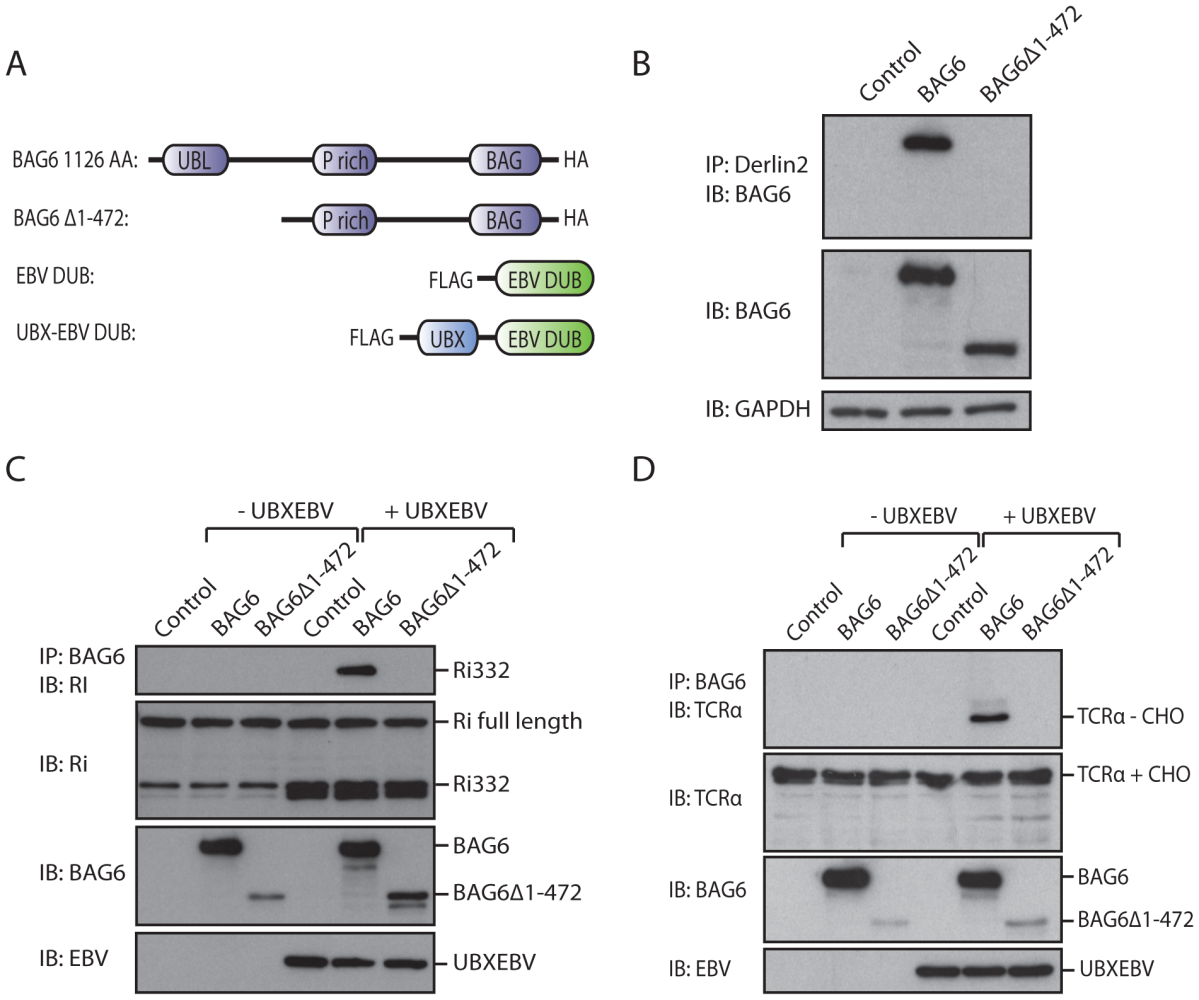
BAG6 binds dislocation substrates via its N-terminal domain. A. Cartoon depicting the BAG6 and EBV DUB constructs used in this study. UBL: Ubiquitin-like domain. P rich: proline-rich domain. BAG: BAG-domain. EBV DUB: catalytic domain of BPFL1. UBX: a p97 interaction domain. B. HEK293 cells were transfected with either BAG6 or BAG6Δ1-472. Cells were lysed in NP40, followed by IP for Derlin2. BAG6 levels were determined by blotting with HA-antibody, where GAPDH serves as a loading control. C. HEK293 cells were transfected with Ri332 (untagged), either BAG6 or BAG6Δ1-472, and in the presence or absence of UBX-EBV. The cells were lysed in NP40, followed by an IP for BAG6 (HA). Ri332 levels were detected using a Ri332 antibody. Input levels of BAG6 and UBX-EBV were detected by immunoblotting with HA and Flag antibodies respectively. D. As in C, except that TCRα is substituted for Ri332.

If established dislocation substrates associate with BAG6 in the cytosol, then we should perhaps be able to use BAG6 to retrieve endogenous dislocation substrates more generally. To this end, we cloned full length BAG6 from total RNA isolated from HEK293T cells (1126 amino acids), and a HA-epitope tag was appended to the C-terminus for detection and retrieval. We engineered a BAG6Δ1-475 deletion mutant as a negative control for our experiments (Figure 1A), as the segment between amino acid 125–475 of BAG6 is important for its association with unstructured polypeptides [10], but did not attempt to narrow down the essential region(s) within this sizable deletion (for detailed analysis for the binding interaction, see [13]). Both constructs are readily expressed in HEK293T cells through transient transfection. The BAG6Δ1-475 construct is expressed at a lower level than the full-length form (Figure 1B). Furthermore, whereas full-length BAG6 is recruited to a protein complex at the ER that contains Derlin2, BAG6Δ1-475 is not (Figure 1B). Instead of using proteasome inhibitors we relied on expression of EBV-DUB to interfere with proteasomal proteolysis, as we have found this method less detrimental to cells for experiments of the type described below [6].

As proof of principle, we examined the recovery of known dislocation substrates in association with BAG6. Either Ri332 or TCRα was expressed in HEK293T cells through transient transfection, in the presence of full length BAG6 or BAG6Δ1-475. Cells were lysed in NP40 lysis buffer, followed by immunoprecipitation of BAG6 via its epitope tag. The recovered complex was eluted in SDS sample buffer, separated by SDS-PAGE and immunoblotted with antiserum against the relevant dislocation substrate. In the absence of EBV DUB co-expression, no substrate was recovered in complex with BAG6, presumably because of ongoing proteasomal degradation. However, upon UBX-EBV DUB co-expression, we retrieved the Ri332 or TCRα substrates in complex with BAG6 (Figure 1C, D). The apparent MW of the retrieved substrates corresponds to the de-glycosylated species, it is this species that accumulates in the cytosol [5], [6], [9]. Cytosolic peptide N-glycanase removes N-linked glycans from the substrate, a reaction used as a diagnostic for cytosolic localization of the substrate [14]. No substrate was retrieved in association with BAG6Δ1-475, indicating that the deleted domain is normally required for interaction between BAG6 and relevant substrates. We can thus retrieve dislocated glycoproteins from the cytosol with full length BAG6.

### BAG6 overexpression does not impair dislocation

**Figure 2:**
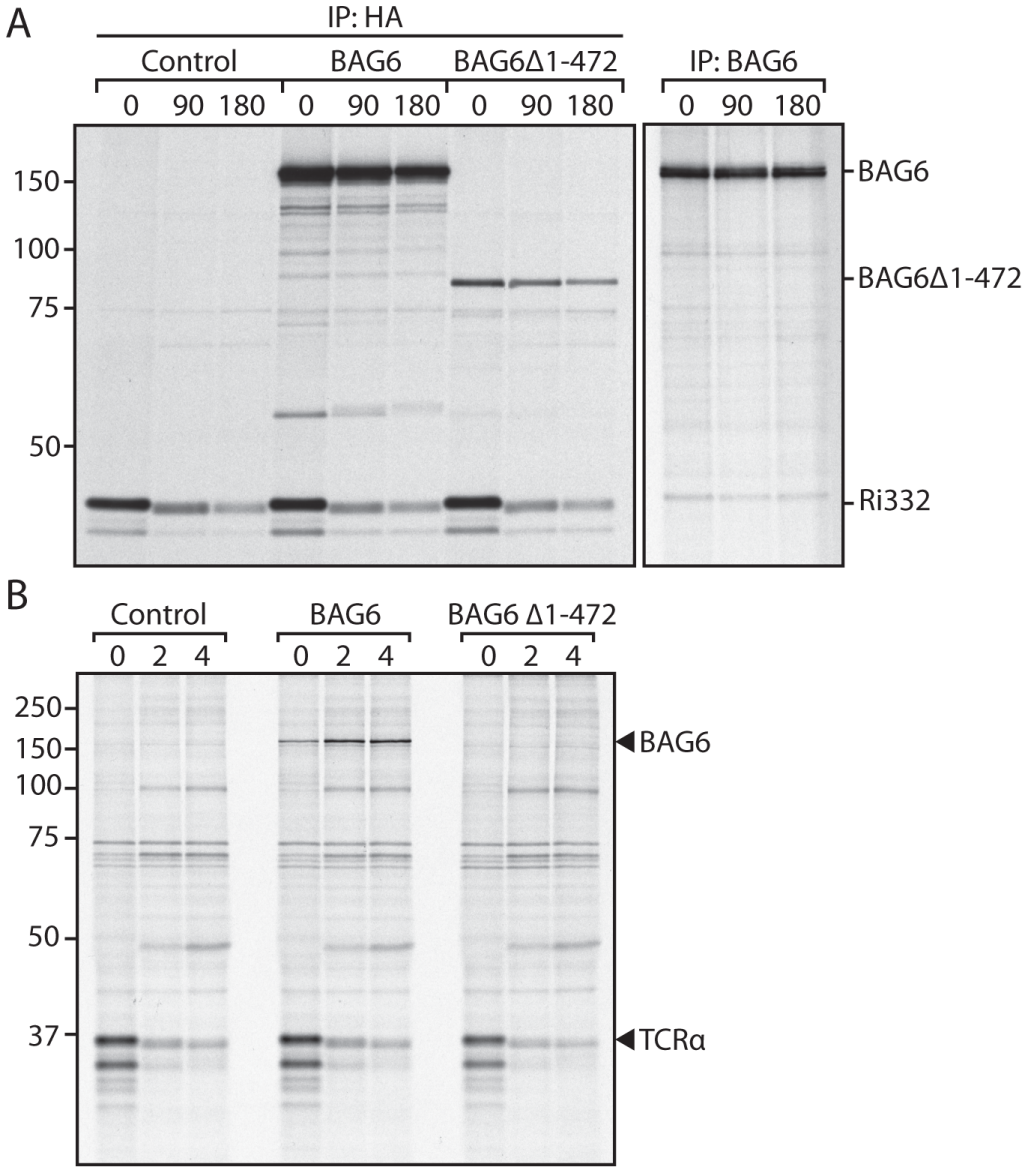
BAG6 expression does not impair dislocation. A. HEK293 cells were transfected with Ri332 and either BAG6, BAG6Δ1-472 or empty vector as a control. The cells were pulse-labeled for 10 min with S35 Cys/Met, after which samples were taken at the indicated chase times. The cells were lysed in 1% SDS to dissolve all protein complexes, diluted in NP40 lysis buffer to 0.1% SDS, followed by IP with HA. The right panel represents untransfected cells pulse-labeled for 1 h with S35 cys/met, after which the individual samples were subjected to IP with BAG6 antibody. B. As in A, except that Ri332 is substituted for TCRα, and the cells were lysed in NP40 lysis buffer.

Overexpression of a protein can disrupt the cellular process in which it participates, even for the wild-type form of the protein, as it might apply here to BAG6. We therefore tested the kinetics of dislocation for both Ri332 and TCRα in cells that overexpress BAG6. Cells were pulse-labeled with ^35^S Cys/Met, after which Ri332 and TCRα were recovered by immunoprecipitation. The kinetics of degradation for Ri332 or TCRα were unaffected by expression of full-length BAG6 or BAG6Δ1-475 (Figure 2A, B). In line with published work, we retrieve a fraction of unglycosylated Ri332, and a fraction of de-glycosylated TCR (6), all of which migrate faster on SDS-PAGE due to its smaller size. BAG6 and Ri332 are both modified with a HA-tag, and can therefore be visualized in a single immunoprecipitation experiment (Figure 2A). In agreement with earlier work, we retrieved BAG6 in association with TCRα (Figure 2B) [5], [9].

### Recovery of endogenous dislocation substrates with BAG6

Having established that we can retrieve known overexpressed dislocation substrates in association with BAG6, can we detect endogenous dislocation substrates in a complex nucleated by BAG6? BAG6 participates in quality control of secretory proteins both prior to their translocation into [12], as well as during their dislocation from the ER. We must therefore distinguish between these two distinct populations of BAG6 clients. Many secretory proteins are modified by N-linked glycosylation, a co-translational modification restricted to the lumen of the ER. When a terminally misfolded protein finds itself in the cytosol N-linked glycans are quickly removed by peptide N-glycanase [15], [16]. By pharmacologically blocking peptide N-glycanase activity, we can separate these two populations -failed translocation, or misfolded in the ER lumen- based on their glycosylation status. For a protein to qualify as an endogenous dislocation substrate, it must therefore associate with BAG6 in the presence, but not the absence of EBV-DUB, and moreover it should be sensitive to the inclusion of an N-glycanase inhibitor. We lack similarly robust criteria to assess dislocation of misfolded non-glycosylated substrates.

HEK293T cells were transiently transfected with UBX-EBV DUB together with empty vector (control), BAG6, or BAG6Δ1-475. Transfectants were cultured for 4 h in the presence of ^35^S Cys/Met to label newly synthesized proteins, and 30 µM Z-VAD-FMK to block peptide N-glycanase [17], a second target of this known Caspase inhibitor. BAG6 and its associated proteins were recovered by immunoprecipitation. One half of the sample was incubated for 2 h at 37°C with PNGaseF, followed by SDS-PAGE. Any PNGaseF-sensitive proteins are considered to be endogenous dislocation substrates that associate with BAG6.

**Figure 3:**
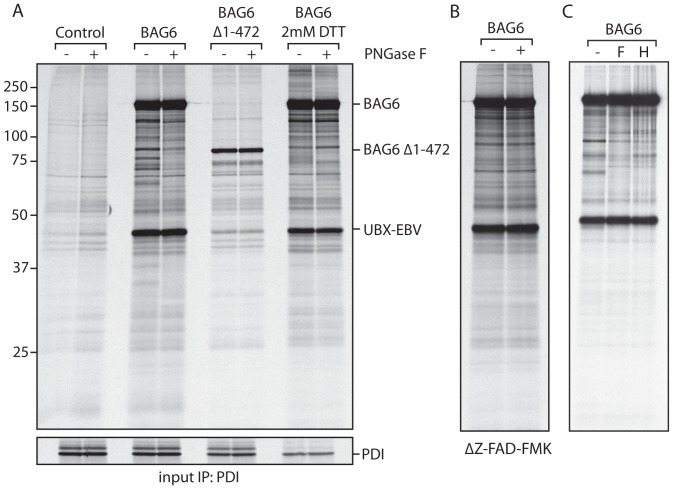
Endogenous dislocation substrates associate with BAG6. A. HEK293 cells were transfected with UBX-EBV and either BAG6, BAG6Δ1-472 or empty vector as a control. The cells were labeled with S35 Cys/Met for 4 h in the presence of 30 µM Z-VAD-FMK, and where indicated 2 mM DTT. The cells were collected, lysed in NP40 lysis buffer, and the samples split in two. All samples were subjected to IP for BAG6 (HA), the retrieved protein eluted and placed at 37°C for 2 h in the presence or absence of PNGaseF. A sequential IP for PDI serves as a loading control, bottom panel. B. As in A, full-length BAG6 only, but in the absence of Z-VAD-FMK. C. As in A, full-length BAG6 only, but the sample was now split in three, and treated with either no enzyme, PNGaseF, or endoH.

**Figure 4:**
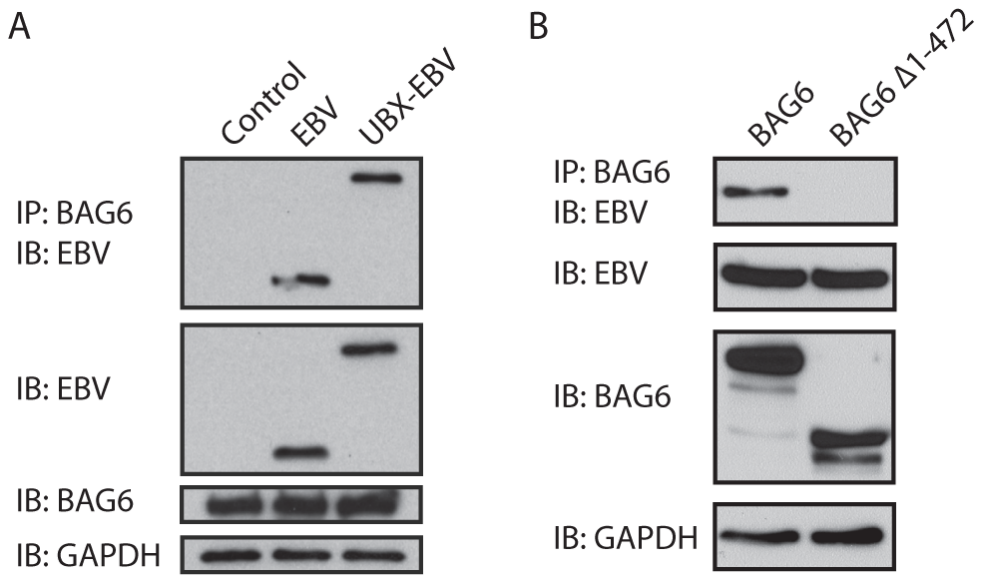
UBX-EBV and BAG6 are found in one protein complex. A. HEK293 cells were transfected with BAG6 and either EBV DUB or UBX-EBV. Cells were lysed in NP40 lysis buffer, and subjected to IP for BAG6 (HA). The eluate was blotted for EBV DUB (FLAG). Input levels of EBV DUB, BAG6 and GAPDH were assessed with the relevant antibodies. B. HEK293 cells were transfected with UBX-EBV and either BAG6 or BAG6Δ1-472. Cells were lysed in NP40, and subjected to IP for UBX-EBV (FLAG). The eluate was blotted for BAG6 (HA). Input levels of UBX-EBV, BAG6 and GAPDH were assessed with the relevant antibodies.

We observed a number of proteins in complex with BAG6 (Figure 3A). Many of these proteins are sensitive to digestion with endoH, indicating the presence of high mannose-type N-linked glycans, as expected for dislocation substrates (Figure 3C). No glycoproteins were retrieved in either the control condition, or in complex with BAG6Δ1-475. PNGaseF sensitive proteins were retrieved only from cells exposed to Z-VAD-FMK, arguing against post-lysis association of misfolded glycoproteins with BAG6 (Figure 3B). We noted a polypeptide of a MW that corresponds to UBX-EBV-DUB after IP for BAG6. As both interact with p97 [6], [9], we tested whether EBV DUB devoid of a p97-interacting UBX-domain still associates with BAG6, which it does (Figure 4A). However, deletion of the N-terminal domain of BAG6 mostly disrupted this interaction (Figure 4B). The mode of association of UBX EBV-DUB and BAG6 was not further investigated.

To raise the concentration of misfolded proteins in the ER, we included a condition in which cells were exposed to 2 mM DTT while the cells were cultured in the presence of ^35^S Cys/Met. DTT is commonly used to induce the UPR, a signaling cascade that is activated to restore homeostasis when the folding capacity of the ER is overwhelmed by accumulation of misfolded proteins. To our surprise, we recovered far fewer glycoproteins in association with BAG6 when the cells were treated with DTT (Figure 3A).

### DTT primarily affects protein synthesis

**Figure 5:**
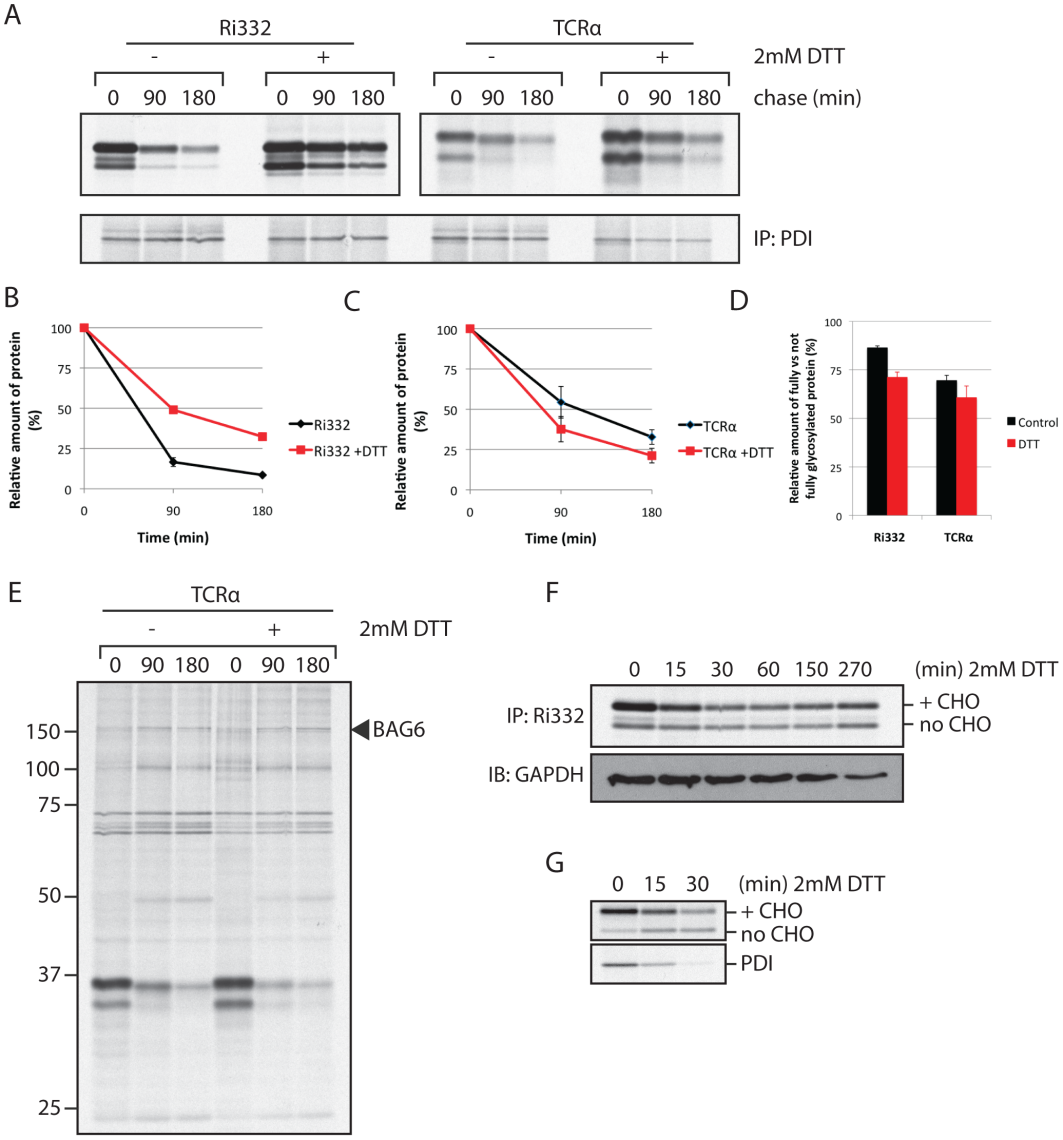
DTT does not significantly halt protein dislocation, but affects translocation into the ER. A. HEK293 cells were transfected with either Ri332 or TCRα, pulse-labeled for 15 min with S35 Cys/Met, after which samples were collected at the indicated chase times. 2 mM DTT was added during the pulse period when indicated. After lysis in 1% SDS, the lysate was diluted in NP40 to 0.1% SDS and subjected to IP for either Ri332 or TCRα. A sequential IP for PDI serves as a loading control. B. Quantification of the degradation of Ri332 as depicted in A (n = 3). C. Quantification of the degradation of TCRα as depicted in A (n = 3). D. Quantification of the relative amount of fully versus not fully glycosylated protein as depicted in A (n = 3). E. HEK293 cells were transfected with TCRα and BAG6. The cells were pulse-labeled for 15 min with S35 Cys/Met, after which samples were collected at the indicated chase times. 2 mM DTT was added during the pulse period when indicated. After lysis in NP40, the samples were subjected to IP for TCRα. F. Cells were transfected with Ri332 and treated with 2 mM DTT for the indicated time period. At the end of the treatment with DTT, the cells were pulse labeled for 10 min with S35 Cys/Met. The cells were lysed in 1% SDS, the lysate was diluted in NP40 to 0.1% SDS and subjected to IP for Ri332 (HA). Inpute lysate was immunoblotted for GAPDH as a loading control. G. As in F, except for a sequential IP for PDI.

Addition of DTT to a cell culture not only impairs oxidative folding of newly synthesized polypeptides in the ER, but also affects ER-resident proteins, including those involved in dislocation. We showed earlier that manipulation of redox potential affects dislocation of Class I MHC products under the agency of HCMV US11 [18]. A number of chaperones linked to dislocation serve as oxidoreductases, thought to maintain polypeptides in a dislocation-competent conformation prior to transfer across the ER membrane [2]. Indeed, when we retrieve protein disulfide isomerase (PDI) by immunoprecipitation from cells exposed to 2 mM DTT, we observe a distinct set of associating proteins (data not shown). We next examined degradation of Ri332 and TCRα as indicators of dislocation. DTT was added to the culture after a 15 min pulse with ^35^S Cys/Met, it was therefor only present during the chase period of the experiment to exclude an effect on protein synthesis (see below). We observed a mild negative effect on the degradation of Ri332, whereas TCRα degradation was in fact slightly faster (Figure 5A–C), from which we conclude that DTT has a limited or no effect on dislocation. A mild defect in N-linked glycosylation was seen in DTT-treated cells: we observed a larger fraction of incompletely N-glycosylated protein (Figure 5A, quantified in 5D). As TCRα normally receives 4 N-linked glycans upon translocation into the ER, a reduction in the number of N-linked glycans could more readily commit TCRα to dislocation. Moreover, we observed a polypeptide with a MW that corresponds to that of BAG6 in association with TCRα, regardless of DTT treatment (Figure 5E) [5].

If dislocation of these two substrates is not affected by treatment with DTT, then why do we not observe more endogenous glycoproteins in association with BAG6? Disruption of ER homeostasis and activation of the UPR cause translational attenuation through phosphorylation of eIF2α [4], and rapid IRE-1 dependent decay of mRNA [19]. Indeed, we observed a rapid decrease of newly synthesized PDI and Ri332 upon treatment with DTT (Figure 5F, G). Expression under the control of a strong promoter, as is the case for Ri332, most likely buffers the effect seen for endogenously expressed proteins such as PDI (Figure 5G), though from this experiment we cannot exclude a specific effect of the DTT treatment on synthesis of PDI. A shortage of new misfolded substrates poised for dislocation could result in our failure to detect glycoproteins in complex with cytosolic BAG6. The observed reduction in protein glycosylation does not seem sufficiently strong to account for the complete failure to retrieve glycoproteins with BAG6, although the effect could be more pronounced for proteins at their endogenous expression level. Alternatively, the experimental conditions could induce a degradation pathway to dispose of misfolded glycoproteins that is independent of BAG6.

### Dislocation as measured by glycoprotein appearance in the cytosol

As shown above, BAG6 interacts in the cytosol with endogenous dislocated glycoproteins, but these likely represent a subset of possible dislocation substrates. We next complemented the BAG6-centered approach with a more general strategy to measure the appearance of glycoproteins in the cytosol. We wondered whether it might be possible, using persisting N-linked glycans that mark dislocated proteins as a retrieval handle, to recover substrates from the cytosol of EBV DUB expressing cells.

Cells were transfected with UBX-EBV DUB, and labeled with ^35^S Cys/Met in the presence of 30 µM Z-VAD-FMK, to allow accumulation of dislocated proteins in the cytosol, with their N-linked glycans still attached [17]. Cells were placed on ice, and incubated with 100 nM perfringolysin-O, a pore-forming toxin that binds cholesterol. After removal of unbound toxin, cells were transferred to 37°C to induce pore-formation, after which the cytosol was separated from the membrane fraction by centrifugation. We previously applied this method to isolate dislocation substrates from the cytosol with minimal contamination of membrane or ER luminal (glyco-) proteins [7].

**Figure 6:**
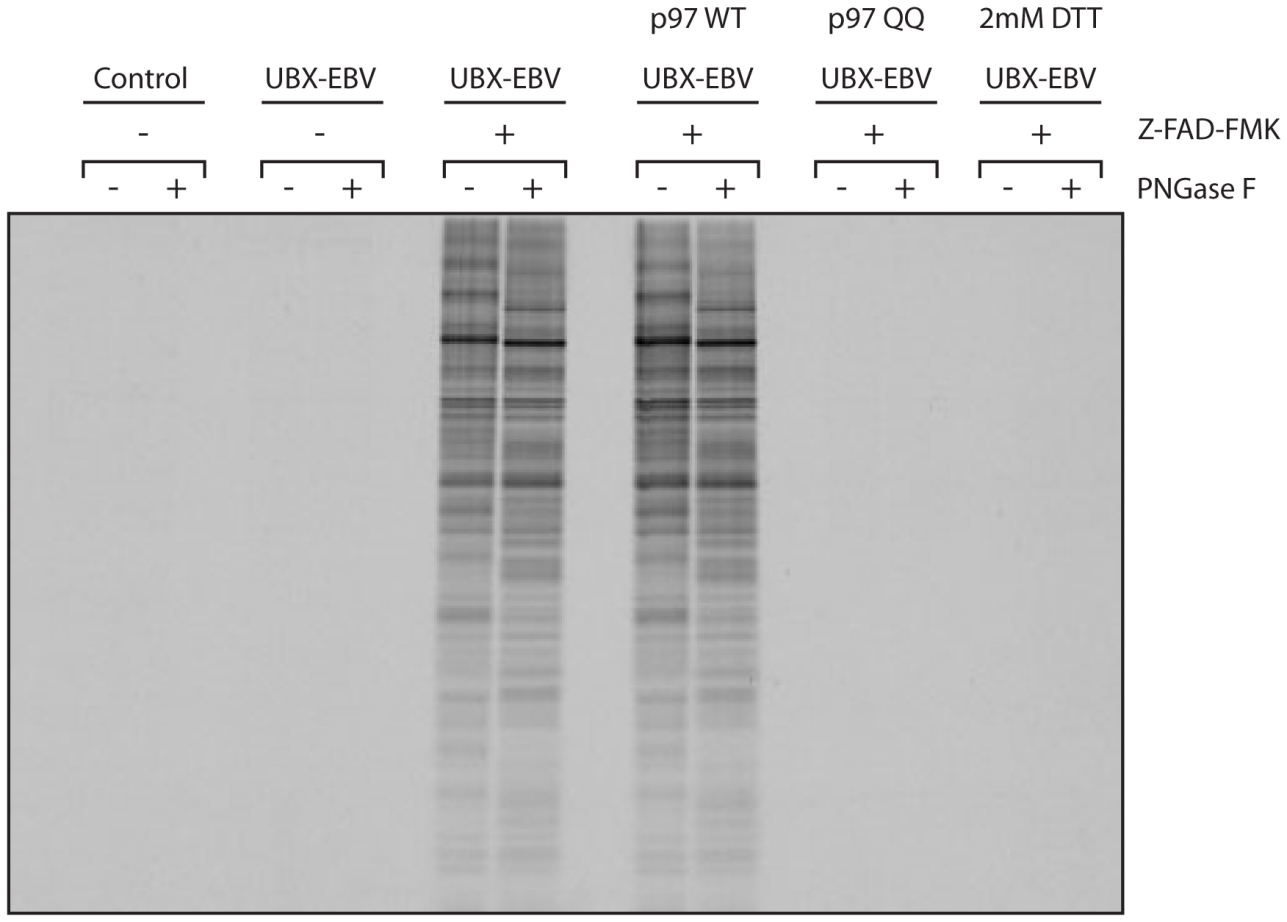
Dislocation can be visualized by the appearance of glycoproteins in the cytosol. HEK293 cells were transfected with either empty vector as a control, or UBX-EBV and/or p97 variants as indicated. The cells were labeled for 4 h with 35S Cys/Met, after which they were collected as a cell pellet and placed on ice. The cells were then resuspended in 100 µl HBSS containing 200 nM PFO. The cells were washed once in cold HBSS, and resuspended in 100 µl HBSS, after which they were transferred to 37°C to induce pore formation. The cells were pelleted for 5 min at 2000 rpm, after which the soluble cytosolic fraction was collected and the pellet discarded. The soluble fraction was dissolved in NP40 lysis buffer, split into two equal parts, and subjected to IP with immobilized Concanavalin A. Retrieved glycoproteins were eluted with mannose and placed at 37°C in the absence or presence of PNGaseF.

The cytosol preparation thus obtained was diluted with a detergent-containing buffer, and incubated with Concanavalin A-agarose beads to recover N-linked glycoproteins. After stringent washing, glycoproteins were eluted with α-methylmannoside. Akin to the experiments with BAG6, we identified dislocated glycoproteins through treatment with PNGaseF after the elution step (Figure 6). No glycoproteins were retrieved from cells that lack EBV-DUB, or that were not exposed to Z-VAD-FMK. The retrieved glycoproteins must therefore be endogenous dislocation substrates. Not all proteins retrieved on ConA-agarose were PNGase F sensitive. These proteins could represent cytosolic factors that associate with misfolded proteins to aid in their degradation, or -less likely- might be the result of incomplete PNGaseF digestion.

If the ConA-agarose retrieved material indeed represents dislocated proteins, a blockade of the dislocation reaction should affect their retrieval. To achieve such a blockade, cells were co-transfected with either wt p97, or p97 QQ, a mutant unable to hydrolyze ATP that acts in dominant-negative fashion to inhibit dislocation. Whereas the protein profile obtained for wt p97 was identical to that seen for the control condition, no glycoproteins were retrieved when p97 QQ was included, confirming what has been stated in numerous publications [20], but only for cherry-picked substrates. A similar result was obtained when cells were treated with DTT.

## Discussion

The ER harbors machinery to control both the quality and the quantity of cargo that traffics along the secretory pathway [21]. Individual misfits are ejected into the cytoplasm -presumably one at a time- to then be cannibalized by the proteasome. More severe perturbations of the ER folding capacity evoke activation of the UPR, which governs influx of client proteins and adjusts the folding capacity of the ER appropriately.

While much is known about the machinery that governs quality control in the ER, far less is known about its endogenous clients. Protein folding is a complex process, and goes hand in hand with co- and post-translational modifications and membrane insertion, where applicable. Information on folding efficiency is anecdotal, in that it must be acquired for each protein individually, as in the case of the polytopic membrane protein CFTR. No general methods are available to measure number or identity of the proteins that fail this barrage [22]. In addition to misfolded products, some signaling molecules are subjected to this pathway as part of their natural signaling cascade (e.g. Insig-1 [23], Hedgehog [24]).

Several known dislocation substrates are engaged by BAG6 once they reach the cytosol. We here show that BAG6 can be used to capture endogenous dislocation substrates in a less biased manner. Substrate engagement is mediated by elements within the N-terminal domain of BAG6, and is not obstructed by the presence of N-linked glycans on the substrate. More precise knowledge on how BAG6 grasps unstructured polypeptides is needed, as it is now firmly implicated in diverse cytosolic pathways that handle unfolded proteins (including DRIPs, tail-anchored proteins, failed translocation products and dislocated glycoproteins [5], [6], [9]–[12]).

The approach chosen here -glycoprotein retrieval under UBX-EBV expression in combination with Z-VAD-FMK- is by no means limited to BAG6: other cytosolic ‘baits’ can presumably be used similarly. We further used a more general approach where N-glycosylated proteins are retrieved on a Concanavalin-A matrix.

This approach has the advantage that it allows a quick sampling of large numbers of dislocation substrates at their endogenous expression level, eliminating the need to test individual dislocation substrates sequentially. We illustrate this through expression of a dominant negative form of p97, which blocks physical extraction of substrates from the ER: in its presence, no glycosylated substrates are retrieved from the cytosol, validating our approach. The expressed constructs and inhibitors used here affect processes centered on the cytosol, thus eliminating the ER itself as the immediate target.

The benefit of studying endogenous dislocation substrates, as distinct from overexpressed proteins, was perhaps most apparent when we treated cells with DTT. Dislocation of newly synthesized Ri332 and TCRα continues in the presence of 2 mM DTT, in line with the requirement for reduction of disulfide bonds to ease transfer through a presumed retro-translocon, the pore diameter of which likely imposes size constraints. Dislocation of Ri332 and TCRα is not affected, and no newly synthesized endogenous glycoproteins appear in the cytosol in the presence of DTT. In line with published work, we observed that protein synthesis is quickly halted upon treatment with DTT. This effect is almost instantaneous and complete; we failed to retrieve newly synthesized glycoproteins from the cytosol of these cells. We conclude that enforced placement of artificial dislocation substrates into a stressed ER may not always yield results that reflect biological events. Overexpression of proteins that can no longer fold, as is common practice to study dislocation, may alter the landscape of the ER and -with it- the outcome of the experiment.
